# A Rapid and Sensitive Loop-Mediated Isothermal Amplification Assay for the Detection of Mulberry Mosaic Dwarf-Associated Virus

**DOI:** 10.3390/bios16090509

**Published:** 2026-09-10

**Authors:** Shaoshuang Sun, Xueping Zhou, Xiuling Yang

**Affiliations:** 1 Shandong Key Laboratory of Wetland Ecology and Biodiversity Conservation in the Lower Yellow River, School of Life Sciences, Qufu Normal University, Qufu 273165, China; sunshaoshuangsss@163.com; 2 State Key Laboratory for Biology of Plant Diseases and Insect Pests, Institute of Plant Protection, Chinese Academy of Agricultural Sciences, Beijing 100193, China; 3 State Key Laboratory of Rice Biology and Breeding, Institute of Biotechnology, Zhejiang University, Hangzhou 310058, China

**Keywords:** mulberry, MMDaV, LAMP, virus diagnosis

## Abstract

Mulberry mosaic dwarf-associated virus (MMDaV), a member of the genus *Mulcrilevirus* in the family *Geminiviridae*, poses a serious threat to mulberry cultivation and growth in China. Early and accurate diagnosis is a prerequisite for the effective prevention and control of MMDaV-related disease. In this study, a loop-mediated isothermal amplification (LAMP)-based detection method was established for the specific identification of MMDaV. Three pairs of specific primers were designed targeting the nucleotide sequences of the MMDaV *V2* gene. The optimized LAMP reaction was performed at a constant temperature of 65 °C for 60 min, and the established method exhibited high specificity with no cross-reactivity observed against other tested geminiviruses. The LAMP method achieved a detection limit of 200 pg of target MMDaV DNA, which was tenfold more sensitive than conventional PCR. Furthermore, the amplification results could be directly visualized via distinct color change. Collectively, the developed LAMP method enables efficient, sensitive, and specific isothermal detection of MMDaV and holds great promise for rapid diagnosis of mulberry viral disease.

## 1. Introduction

Plant viruses are obligate intracellular parasites that account for nearly half (47%) of all emerging plant disease epidemics, causing substantial economic losses worldwide each year [1,2,3]. With the development of small RNA-based deep sequencing, an increasing number of plant viral species have been identified. However, no effective chemical agents are currently available to cure plant viral diseases. Effective management of plant viruses therefore relies on integrated strategies, including strict quarantine, cultivation of virus-free seedlings, and deployment of virus-resistant crop cultivars [4]. Accordingly, early and accurate diagnosis and detection of virus is a fundamental prerequisite for limiting the spread of plant viruses. As plants infected with viruses usually show symptoms such as stunted growth and development, yellowing and mosaic leaves, and deformed and necrotic fruits [5], symptom observation has long been the most accessible initial approach for diagnosis. However, symptom development requires a latent infection period in host plants, and mixed infection with different viruses is common under natural conditions, enhancing the complexity of visual diagnosis. Moreover, distinct viral pathogens can induce highly similar symptoms on the same host species. Therefore, reliable and accurate detection tools are essential for precise diagnosis and effective management of plant viral diseases.

Polyclonal or monoclonal antibody-based serological methods offer significant advantages in ease of manipulation, high-throughput screening, and cost effectiveness for large-scale detection of plant viruses. However, their application depends on the availability of specific and high-quality antibodies, which remain unavailable for many plant viruses [6]. Nucleic acid-based detection methods offer an alternative solution to circumvent these limitations. Polymerase chain reaction (PCR) and its derivatives have revolutionized plant virus detection by providing superior sensitivity and specificity through amplification of target viral genomic sequences. Nevertheless, conventional PCR requires highly specialized personnel and thermal cycling equipment, which restricts its use in field or resource-limited settings [7]. In recent years, isothermal nucleic acid amplification techniques have emerged as promising alternatives to PCR, enabling viral nucleic acid amplification under constant-temperature conditions [8,9]. Among these, loop-mediated isothermal amplification (LAMP) is one of the most widely adopted diagnosis technologies and has opened new avenues for plant virus diagnosis [10]. The LAMP reaction employs the auto-cycling and high DNA strand displacement activity of the *Bst* polymerase and a set of four to six primers that specifically recognize six to eight different regions of the target gene, thereby facilitating rapid and specific nucleic acid amplification at a constant temperature ranging from 60 to 65 °C [11,12,13]. To date, LAMP has been implemented in the fields of medicine, agriculture, and food industries for diverse applications, including pathogen detection, mutation screening, and genetically modified organism monitoring [14,15,16,17,18].

White Mulberry (*Morus alba*) is an economically important tree species that has been cultivated in China for thousands of years. Its leaves serve as the exclusive feed source for silkworms, and the berries are rich in antioxidants, vitamins, and minerals. Nevertheless, mulberry production is increasingly threatened by diverse viruses, which pose a significant threat to both yield and quality. Mulberry mosaic dwarf-associated virus (MMDaV), a monopartite geminivirus isolated from mulberry trees showing mosaic and dwarfing symptoms in China, represents a critical emerging viral pathogen of mulberry. MMDaV encompasses a circular single-stranded DNA genome of 2952 nucleotides (nts). It shares low sequence similarity to previously identified geminiviruses, and is therefore assigned to the genus *Mulcrilevirus* of the family *Geminiviridae* [19]. The viral strand of MMDaV encodes five open reading frames (ORFs, namely *V1*, *V2*, *V3*, *V4*, and *V5*), and the complementary strand encodes two ORFs (C1 and C1:C2) [20]. The *V2* protein functions as a suppressor of RNA silencing, and its nuclear localization is essential for its silencing suppression activity. Moreover, the RepA protein facilitates the nucleolar exclusion of *V2* and triggers cell death in *Nicotiana benthamiana* leaves in a WRKY1-dependent manner [21,22]. Despite these insights, current knowledge on MMDaV remains considerably less comprehensive than that of well-characterized begomoviruses or curtoviruses within the same family.

In this study, a precise, sensitive, and rapid LAMP detection method was established for MMDaV. The entire LAMP diagnostic procedure can be completed within one hour using only a simple heating block at 65 °C, with positive amplification readily distinguishable via SYBR Green I-mediated color changes. Owing to its simplicity and dependability, this method provides a practical basis for rapid field diagnosis of MMDaV.

## 2. Materials and Methods

### 2.1. Virus Sources and Plant Materials

Mulberry samples showing upward leaf curling, mosaic, and dwarf symptoms were collected from Ankang County, Shaanxi Province, China.

Viral infectious clones used in the experiment, including tomato yellow leaf curl China virus (TYLCCNV, AJ319675) [23], tomato yellow leaf curl virus (TYLCV, MN432609) [24], tomato leaf curl Yunnan virus (TbLCYnV, AJ971265) [25], tomato golden mosaic virus (TGMV) [26], and beet severe curl top virus (BSCTV) [27], were maintained in our laboratory. *Agrobacterium tumefaciens* EHA105 containing each viral infectious clone was individually adjusted to an optical density of OD_600_ = 1.0 with infiltration buffer (10 mM MgCl_2_, 10 mM MES, pH 5.6, and 100 μM acetosyringone). Four-week-old *N. benthamiana* plants were used for agroinfiltration as previously described [28]. Plants were grown in an insect-free greenhouse at 25 °C under a 16 h light/8 h dark photoperiod.

### 2.2. DNA Extraction and PCR

Total DNA was isolated from virus-infected newly emerging leaves or healthy plant leaves using the cetyltrimethylammonium bromide (CTAB) method [29]. DNA extracted from 0.1 g of each sample was eluted with 50 μL of preheated ddH_2_O. The concentration and purity of the DNA were assessed using a NanoDrop™ One spectrophotometer (Thermo Scientific, Wilmington, DE, USA), and the samples were stored at −30 °C for further analysis. Viral infection was confirmed by PCR detection of viral DNA using 2 × Easy Taq Super Mix (Transgene, Beijing, China) using primers listed in Table S1. The resulting PCR products were analyzed by electrophoresis on a 1.5% agarose gel.

### 2.3. Design of LAMP Primers

**Figure 1:**
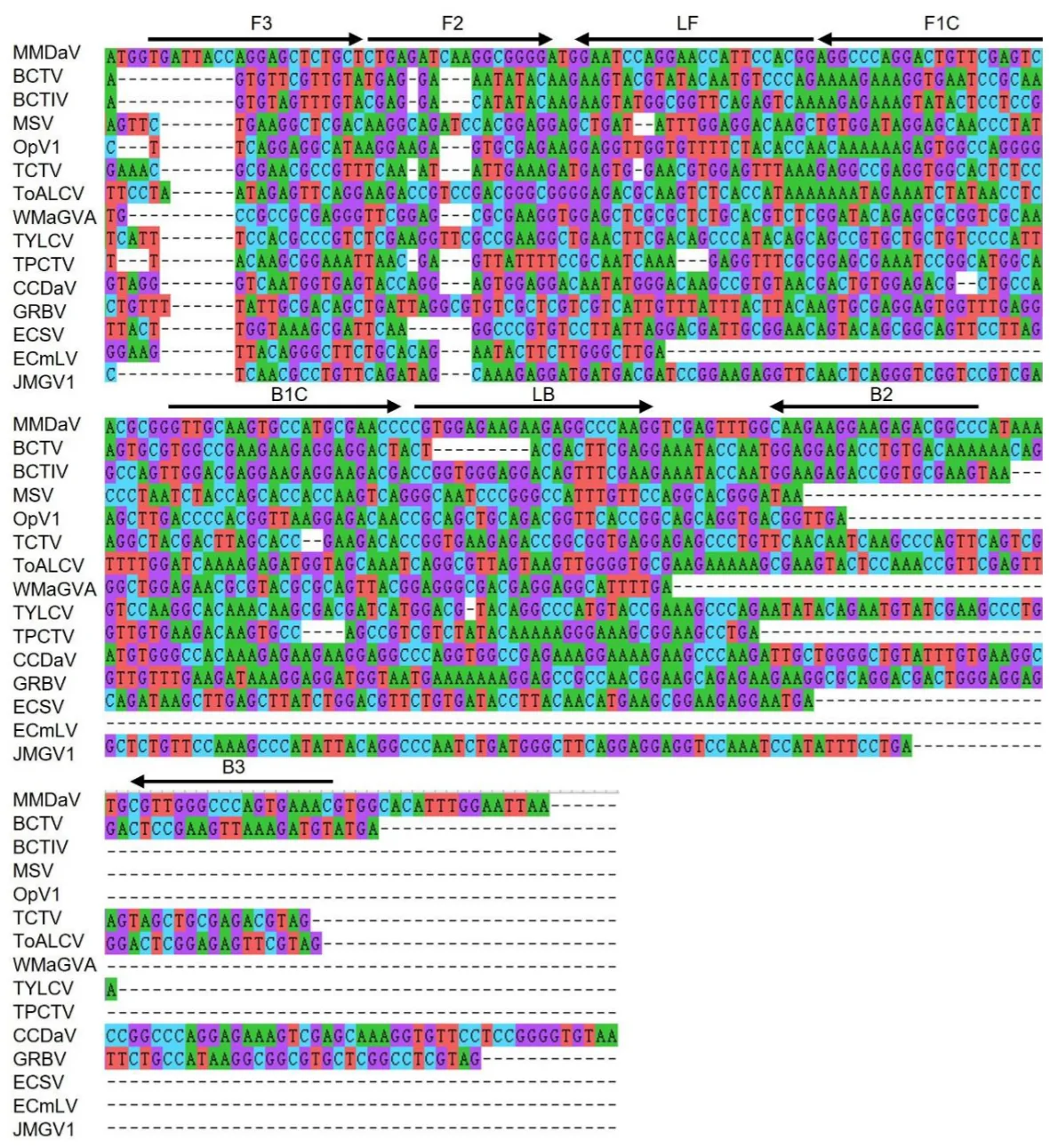
**Primer design for the MMDaV LAMP assay.** Alignment of the nucleotide sequences of the MMDaV *V2* gene with homologous sequences from representative viruses covering 14 genera of the family *Geminiviridae*. The geminiviruses used for sequence alignment are listed in Table S2. Black arrows indicate the binding positions and corresponding names of the LAMP primers used in this study. The FIP primer comprises the F1C and F2 regions, while the BIP primer consists of the B1C and B2 regions.

The complete genome sequence of MMDaV (GenBank accession number KP728254) was compared to selected geminiviruses using the Molecular Evolutionary Genetics Analysis software (https://www.megasoftware.net (accessed on 16 March 2025)) (Figure 1, Table S2). LAMP primers were designed targeting the MMDaV *V2* using the Primer Explorer Software (http://primerexplorer.jp/lampv5e/index.html (accessed on 9 April 2025)). The positions and sequences of the LAMP primers used in this study are shown in Figure 1 and Table S1, including two inner primers (FIP: 5′-GACTCGAACAGTCCTGGGCCT-CTGAGATCAAGGCGGGGA-3′ and BIP: 5′-GTTGCAAGTGCCATGCGAACC-GGCCGTCTCTTCCTTCTTG-3′), two outer primers (F3: 5′-TGATTACCAGGAGCTCTGCT-3′ and B3: 5′-GTTTCACTGGGCCCAACG-3′), and two loop primers (LF: 5′-CCGTGGAATGGTTCCTGGATTC-3′ and LB: 5′-CGTGGAGAAGAAGAGGCCCAAG-3′). In parallel, the *V2* gene sequence of the reference MMDaV isolate (GenBank accession number KP728254) was aligned with all available MMDaV sequences retrieved from NCBI (Table S3). For in silico specificity assessment, the LAMP primer set targeting *V2* was used as queries against the genomic sequences of selected mulberry-infecting viruses, including mulberry vein banding virus (NC_026619, S segment; NC_026618, M segment; NC_026617, L segment), mulberry mosaic leaf roll-associated virus (NC_038767, RNA1; NC_038768, RNA 2), and mulberry badnavirus 1 (NC_026020).

### 2.4. Optimization of Reaction Conditions for the LAMP-Based Detection of MMDaV

The LAMP assay was performed in a 25 μL reaction mixture containing the following components: 2.5 μL of 10 × Isothermal Amplification Buffer, 1.5 μL of MgSO_4_ (100 mM), 3.5 μL of dNTPs (10 mM), 4 μL of betaine (5 M) (Sigma, St. Louis, MO, USA), 4 μL of FIP and BIP (10 μM), 0.5 μL of F3 and B3 (10 μM), 1 μL of LF and LB (10 μM), 1 μL of *Bst* WarmStart DNA Polymerase (8000 U/mL, New England Biolabs, Ipswich, MA, USA), 2 μL of DNA template, and 5 μL of ddH_2_O. To optimize the reaction conditions, amplification was performed at a gradient of temperatures ranging from 60 °C to 69 °C (set by the thermocycler: 60, 60.9, 62.1, 63.6, 65.7, 67.3, 68.4, 69 °C) and over different incubation periods (10, 20, 30, 40, 50 and 60 min). After incubation, 0.25 μL of 10,000 × SYBR Green I (Solarbio, Beijing, China) was added to each reaction tube for visual detection of amplification products. The amplicons were separated by electrophoresis on a 1.5% agarose gel. Fragments of about 200 bp were excised and purified using the E.Z.N.A.Gel Extraction Kit (Omega, Norcross, GA, USA). The purified fragment was cloned into the pEasy-Blunt Simple Cloning vector (Transgene, Beijing, China), and the positive clone was selected and sequenced. The resulting sequence was aligned against the reference MMDaV *V2* sequence (GenBank accession number KP728254) using the Molecular Evolutionary Genetics Analysis software (https://www.megasoftware.net (accessed on 16 May 2025)). All experiments were performed in triplicate.

### 2.5. Sensitivity of the LAMP Assay

The sensitivity of the optimized LAMP assay was compared with that of conventional PCR using 10-fold serial dilutions of total DNA (ranging from 200 ng to 20 fg) extracted from MMDaV-infected mulberry leaves. The amplification products of the LAMP assay were analyzed both by visual inspection of color change and by electrophoresis on a 1.5% agarose gel to verify the characteristic ladder-like band. Conventional PCR products were analyzed by agarose gel electrophoresis for the presence of a specific band of the expected size. All experiments were performed in triplicate.

### 2.6. Specificity of the LAMP Assay

The specificity of the LAMP assay was evaluated using total DNA (200 ng per reaction) extracted from mulberry leaves infected with MMDaV and *N. benthamiana* leaves infected with other geminiviruses, including TYLCCNV, TYLCV, TLCYnV, TGMV and BSCTV. DNA extracted from healthy mulberry or *N. benthamiana* plants was used as negative controls. All reactions were performed at 65 °C for 60 min, and each experiment was carried out in triplicate.

## 3. Results

### 3.1. Establishment and Optimization of Reaction Conditions for LAMP Detection of MMDaV

Primer design is critical for ensuring efficient and specific amplification in LAMP assay. Initially, multiple LAMP primer sets were designed to target different ORFs of MMDaV. Key parameters, including primer length, GC content, melting temperature (Tm), stem-loop structural stability, and inter-primer complementarity, were comprehensively evaluated. Two candidate primer sets targeting the *V1* and *V2* genes were selected for subsequent experimental validation. However, the primer set targeting the *V1* gene generated false-positive signals, whereas the *V2*-targeting primer set produced specific amplification and was used for subsequent studies (Figure 1).

**Figure 2:**
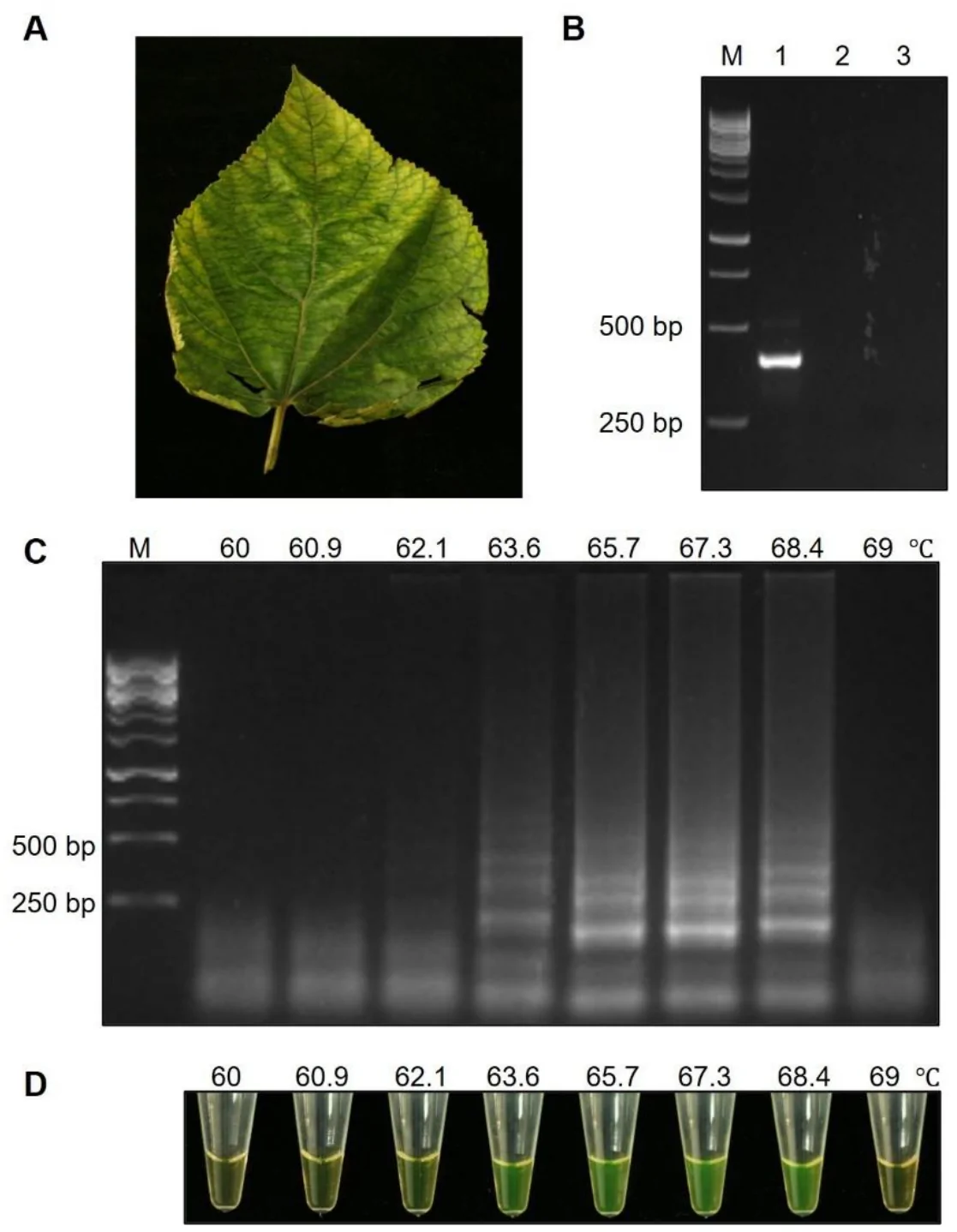
**Establishment of the LAMP assay for MMDaV detection.** (**A**) Symptomatic mulberry leaves exhibiting typical symptoms of MMDaV infection. (**B**) PCR detection of MMDaV using primer pairs MCP746f/MCP1148r. Lane M, 1 kb DNA ladder; Lane 1, DNA extracted from symptomatic mulberry leaf; Lane 2, DNA extracted from healthy mulberry leaf; Lane 3, ddH_2_O. (**C**) Agarose gel electrophoresis of LAMP products amplified at different temperatures. (**D**) Visualization of LAMP products via SYBR Green I staining. The temperatures used for LAMP are shown as indicated. Each experiment was repeated three times.

To determine the optimal reaction conditions, a symptomatic mulberry leaf sample was first confirmed to be infected with MMDaV by PCR and sequencing (Figure 2A,B), and the DNA extracted from this sample was used as the template for subsequent optimization assays. The LAMP reaction was conducted across a temperature gradient ranging from 60 °C to 69 °C for 60 min. Amplification outcomes were evaluated based on the ladder-like bands on agarose gels and color change from reddish-brown to green upon addition of SYBR Green I. No amplification products were detected at 60–61 °C. Faint ladder-like bands and a slight color change started to appear at 62 °C. Amplification efficiency increased markedly with elevated temperature and peaked at 67 °C, as evidenced by the most intense ladder-like bands and distinct green fluorescence. By contrast, amplification was inhibited at 69 °C (Figure 2C,D). Although the highest amplification efficiency was observed at 67 °C, 65 °C was selected as the optimal reaction temperature to achieve a balance between amplification efficiency and assay robustness while minimizing the risk of non-specific amplification triggered by high temperature and ensuring good inter-batch reproducibility.

**Figure 3:**
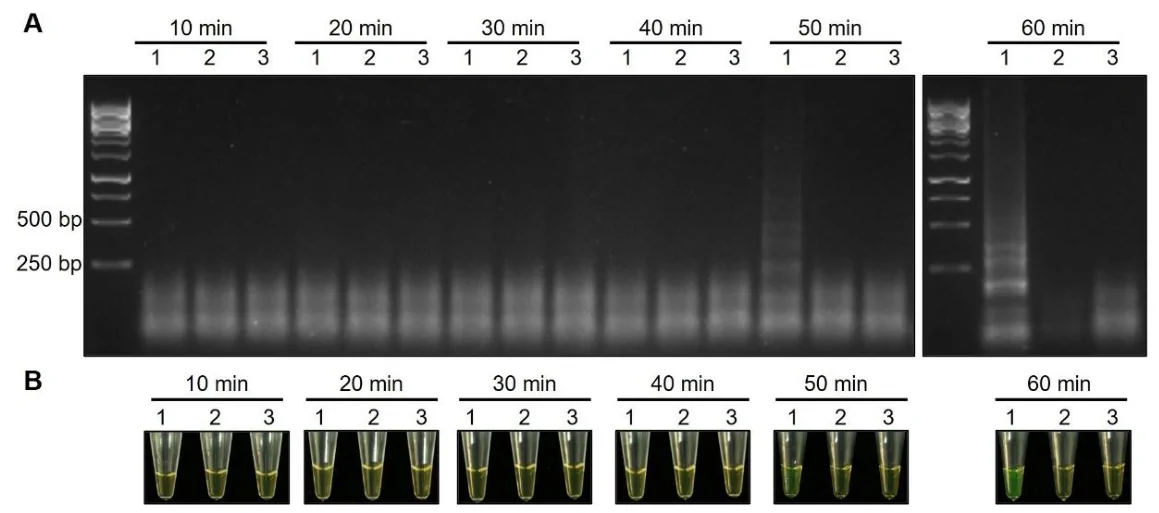
**Optimization of reaction time for the MMDaV LAMP assay.** (**A**) Agarose gel electrophoresis of LAMP products amplified with different reaction time. (**B**) Visual detection of LAMP products amplified under different reaction time. Lane 1 and lane 2 represent DNA extracted from MMDaV-infected or healthy mulberry leaf, respectively. 3 represents ddH_2_O. All experiments were repeated three times.

We further evaluated the effect of reaction time (10–60 min) on LAMP amplification efficiency. No detectable amplification products were observed within the first 50 min of incubation. Positive amplification signals became readily visible after 50 min, and extended incubation to 60 min significantly enhanced the intensity of ladder-like bands and produced a more prominent green fluorescence upon SYBR Green I staining (Figure 3). By contrast, no amplification signal was detected in either the healthy mulberry control or the no-template control. Therefore, 60 min was defined as the optimal reaction time for the MMDaV LAMP assay.

### 3.2. Sensitivity of the LAMP Assay for MMDaV Detection

**Figure 4:**
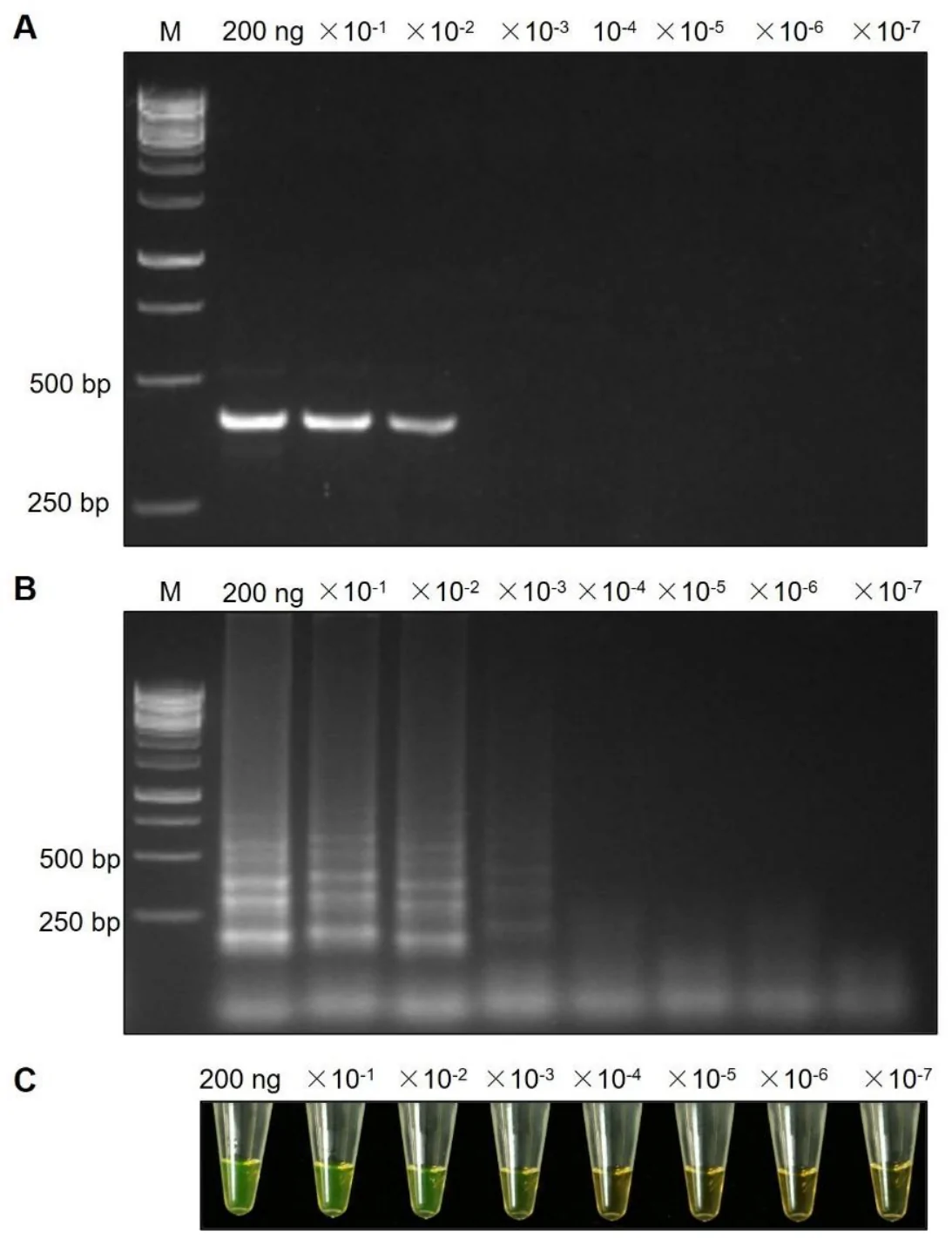
**Sensitivity of PCR- and LAMP-based assay for MMDaV detection.** (**A**) Agarose gel electrophoresis of PCR amplicons generated from serial diluted template DNA. (**B**) Agarose gel electrophoresis of LAMP products using different concentrations of the template DNA. (**C**) Visual detection of LAMP products using different concentrations of the template DNA. All experiments were repeated three times.

To compare the sensitivity of the MMDaV LAMP assay with that of conventional PCR, a tenfold serial dilution assay was performed. Total genomic DNA extracted from MMDaV-infected mulberry leaves was serially diluted in 10-fold gradients, with concentrations ranging from 200 ng to 20 fg per reaction, and parallel detections were conducted using both amplification methods. The amplification products of the two assays were analyzed via agarose gel electrophoresis and SYBR Green I staining. Both the LAMP and PCR assays generated detectable amplicons at the 1000-fold dilution (2 ng of template DNA per reaction). At the 10,000-fold dilution (200 pg of template DNA per reaction), the LAMP assay consistently yielded characteristic ladder-like bands and positive green fluorescence; whereas conventional PCR failed to generate any detectable amplicon at this concentration (Figure 4). Based on this definitive cut-off, the detection limit of the LAMP assay was determined to be 200 pg of total DNA per reaction, whereas that of conventional PCR was 2 ng total DNA per reaction. These results indicate that the sensitivity of the LAMP assay is tenfold higher than that of conventional PCR for MMDaV detection.

### 3.3. Specificity of the LAMP Assay for MMDaV Detection

**Figure 5:**
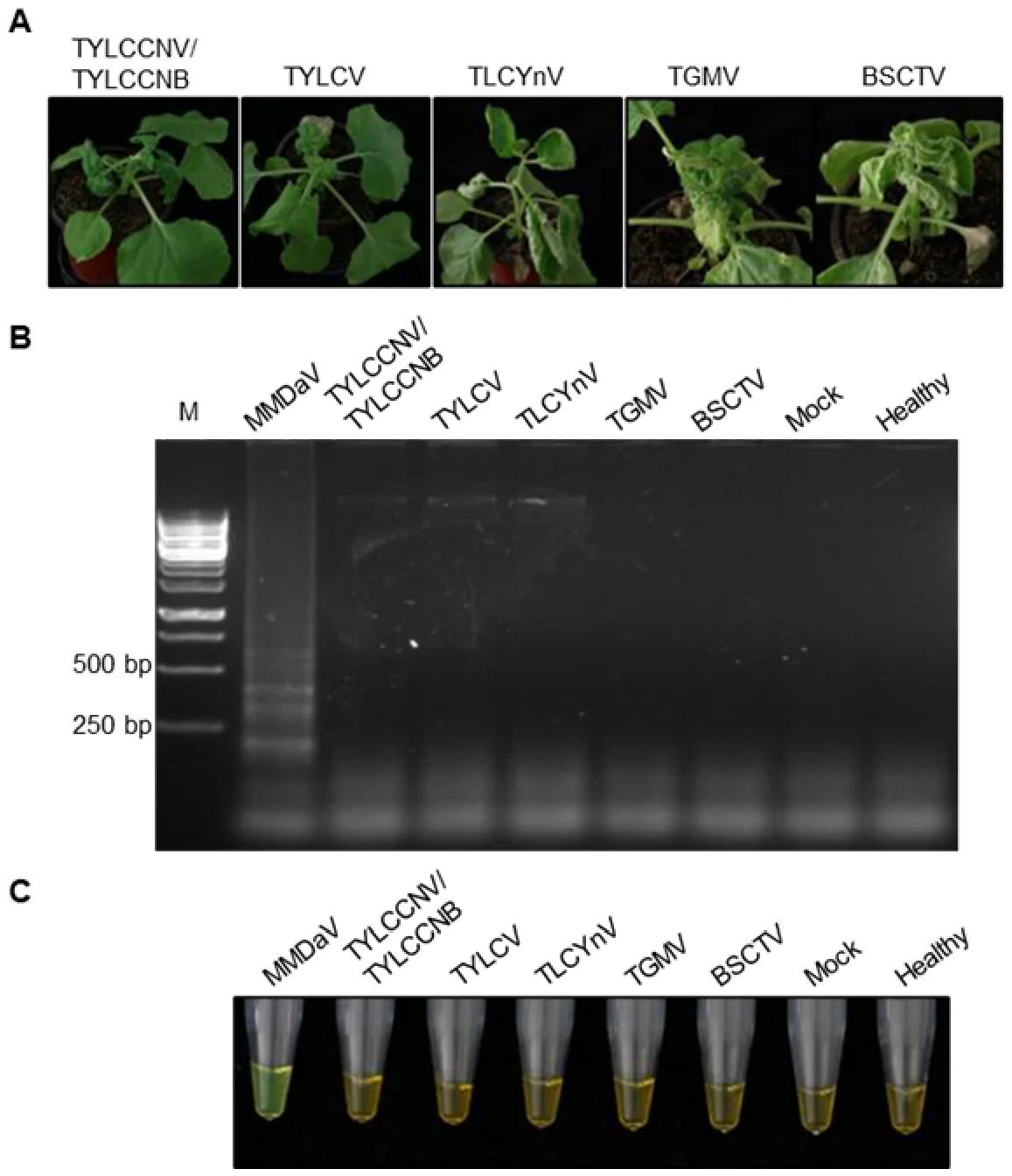
**Specificity analysis of the optimized MMDaV LAMP assay.** (**A**) Symptoms of plants infected with different geminiviruses. (**B**) Agarose gel electrophoresis of LAMP amplicons using DNA isolated from different geminivirus-infected leaf tissues as the template. (**C**) SYBR Green I-based visual detection of LAMP products from different DNA samples. All experiments were repeated three times.

The specificity of the established MMDaV LAMP assay was evaluated using total DNA extracted from MMDaV-infected mulberry leaves and from *N. benthamiana* plants infected with four distinct begomoviruses (TYLCCNV, TYLCV, TLCYnV, and TGMV) and a curtovirus (BSCTV) (Figure 5A). Characteristic ladder-like bands indicative of LAMP amplification were exclusively detected in MMDaV-infected mulberry samples. By contrast, no specific amplification products were detected in samples infected with other tested geminiviruses or in the negative controls (Figure 5B). Consistent results were further validated via SYBR Green I staining, which showed positive green fluorescence only in MMDaV-positive reactions (Figure 5C). These results demonstrated that the optimized LAMP assay is highly specific for MMDaV detection, with no cross-reaction with other tested geminiviruses.

### 3.4. Validation of LAMP Assay for MMDaV Detection

**Figure 6:**
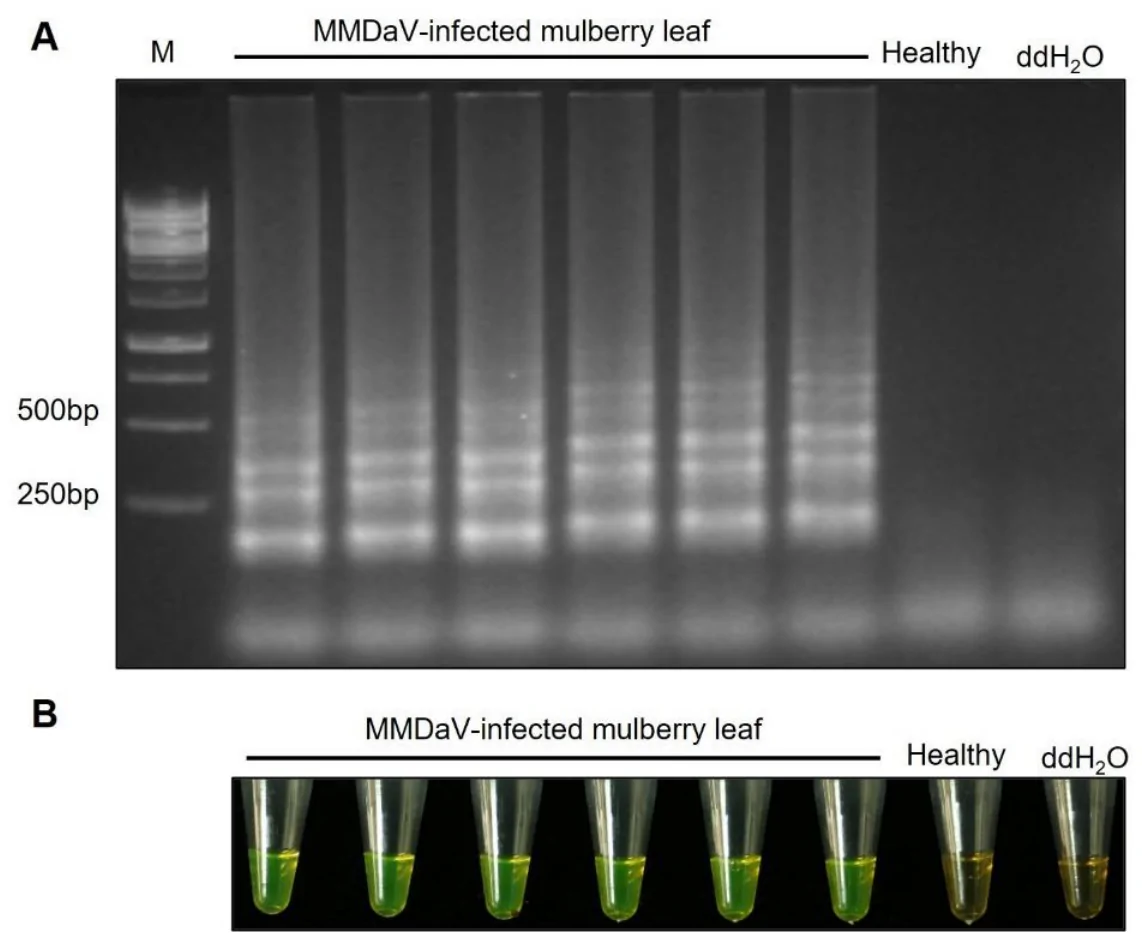
**Detection of MMDaV in mulberry leaves collected from the field.** (**A**) Agarose gel electrophoresis of LAMP products using DNA extracted from different field samples as the template. (**B**) SYBR Green I-mediated visual detection of LAMP products. LAMP assays were conducted at 65 °C for 60 min.

To validate the feasibility of the LAMP assay for field detection of MMDaV, seven mulberry leaf samples collected from natural field conditions were tested for MMDaV. All six symptomatic field samples produced characteristic ladder-like amplification bands on agarose gels and displayed positive green fluorescence after SYBR Green I staining (Figure 6). In contrast, no amplification signal was detected in either the healthy mulberry controls or the no-template blank controls (Figure 6). The specificity of the amplified products was further confirmed by sequencing the purified bands of approximately 200 bp, which was determined to be 145 bp and shared 97% nucleotide sequence identity with the reference MMDaV sequence (GenBank accession number KP728254). PCR-based detection verified the presence of MMDaV in all six symptomatic leaf samples (data not shown). These results demonstrate that the developed LAMP assay is a robust, specific, and reliable tool for the accurate detection of MMDaV in field-collected samples.

## 4. Discussion

To meet the demand for early and rapid diagnosis of MMDaV, this study developed and optimized a reliable LAMP-based detection tool that enables rapid, specific, and sensitive detection of MMDaV under isothermal conditions, eliminating the dependency on thermocyclers and highly trained personnel associated with conventional PCR. The LAMP assay established in this study offers several significant advantages. First, it exhibits high specificity, capable of accurately distinguishing MMDaV from other representative geminiviruses with no cross-reaction. Second, the assay achieves ten-fold higher sensitivity than conventional PCR. Third, consistent detection performance with field-collected mulberry samples validated its robustness and practical applicability for on-site testing. Therefore, this optimized method represents a promising molecular tool for field surveillance and early warning of MMDaV.

Designing an appropriate primer set is critical for the success and specificity of any nucleic acid-based detection assay [30]. In this study, preliminary primers targeting the *V1* gene of MMDaV enabled complete amplification within 30 min but generated false-positive signals (data not shown). This is likely attributable to the sequence architecture of the sequences of MMDaV *V1*, which contains widespread short repetitive oligonucleotides, AT-rich segments, and frequent palindromic motifs. These structural features might promote primer dimerization and random mismatches under isothermal conditions, leading to template-independent false amplification. Collectively, alignment of the representative genomes from all 14 established genera within the family *Geminiviridae*, together with a comparative analysis of all full-length MMDaV genomes available in GenBank, demonstrated that the *V2*-targeting LAMP assay exhibits high species specificity and that the primer-binding regions are highly conserved among all MMDaV isolates. Following optimization of reaction parameters, the *V2*-targetingprimer set, combined with incubation at 65 °C for 60 min, was determined to be the optimal condition. This optimized system ensures high amplification efficiency and stable performance, thereby guaranteeing accurate and reproducible detection of MMDaV.

Specificity and sensitivity are two important parameters in the development of a novel detection method. In this study, no non-specific amplification was observed in samples infected with other representative geminiviruses (TYLCCNV/TYLCCNB, TYLCV, TLCYnV, TGMV, and BSCTV) or in negative controls, confirming the high specificity of the established MMDaV LAMP assay in distinguishing MMDaV from other geminiviruses. Although cross-reactivity tests against other mulberry-infecting viruses, mulberry vein banding virus, mulberry mosaic leaf roll-associated virus, and mulberry badnavirus 1 have not yet been evaluated due to the unavailability of these viral resources, two lines of evidence suggest that the risk of cross-reactivity of these viruses is minimal. First, the former two viruses possess RNA genomes whose amplification requires reverse transcription, whereas MMDaV is a DNA virus; the LAMP assay developed in this study employs DNA as the amplification template without a reverse transcription step, thereby precluding the amplification of RNA viral genomes. Second, in silico analysis revealed no potential binding sites for the *V2*-targeting primer set in the genome sequences of the three viruses. Although the absolute quantification using copy numbers per reaction is widely regarded as the gold standard for assessing the analytical sensitivity of nucleic acid amplification assays, we deliberately employed the total DNA input as the metric in this study, primarily considering the practical applicability of the method in potential field diagnostics, the ultimate goal of which is to enable direct detection of MMDaV in crude or minimally processed field samples. Compared to conventional PCR, the LAMP assay achieves a detection limit of 0.2 ng total DNA per reaction, which is at least ten-fold more sensitive than conventional PCR. This sensitivity is comparable to that of previously reported LAMP detection methods for other geminiviruses, such as tomato leaf curl New Delhi virus (ToLCNDV) and TYLCV [31,32]. Unlike quantitative real-time PCR, which requires costly fluorescent probes or sophisticated instrument for data acquisition, the LAMP assay enables endpoint detection via simple colorimetric readout, substantially reducing both instrumental and operational costs. Moreover, LAMP was shown to exhibit stronger tolerance to plant-derived inhibitory substances, such as phenolic compounds, tannins, and complex polysaccharides relative to conventional PCR [33]. Overall, our established LAMP assay achieves a favorable balance among equipment requirement, detection sensitivity, and experimental cost.

Several limitations of the developed LAMP method should be acknowledged. First, only seven field samples were used to evaluate the practical applicability of this method, owing to the limited availability of field-collected samples. A larger-scale validation with a broader sample set is therefore required to further assess its diagnostic performance under diverse field conditions. Second, the limit of detection was quantified using crude total DNA extracted from infected plant tissues rather than using absolute quantification of the copy numbers of viral genome, which makes it difficult to directly compare the sensitivity of our assay with that of commonly used method such as quantitative PCR. Third, due to the lack of an artificial infection system for MMDaV, it remains unclear how early the developed LAMP assay can detect the virus before symptom onset. Fourth, SYBR Green I was added after the LAMP reaction to avoid its inhibitory effect on isothermal amplification, which increases the potential risk of releasing amplification-containing aerosols, leading to cross-contamination. To minimize false-positive signals arising from carryover contamination, we implemented strict laboratory practices, including physical separation of pre- and post-amplification areas, use of filter-tipped pipettes, and routine decontamination of work surfaces.

In conclusion, the novel LAMP assay established in this study exhibits distinct advantages over current PCR-based detection techniques, including simplicity, high efficiency, specificity, and sensitivity, and easy visualization of readout. It lays a foundation for future point-of-care diagnostics of mulberry mosaic dwarf-associated disease.

## Data Availability

The original contributions presented in this study are included in this article; further inquiries can be directed to the corresponding authors.

## Supplementary Materials

The following supporting information can be downloaded at: https://www.mdpi.com/article/10.3390/bios16090509/s1, Table S1: Primers used in this study. Table S2: Geminiviruses used for sequence alignment in Figure 1; Table S3: MMDaV isolates used for alignment of *V2* nucleotide sequences.

## Abbreviations

The following abbreviations are used in this manuscript:

MMDaV	Mulberry mosaic dwarf-associated virus
LAMP	Loop-mediated isothermal amplification
ORFs	Open reading frames
TYLCCNV	Tomato yellow leaf curl China virus
TYLCV	Tomato yellow leaf curl virus
TbLCYnV	Tomato leaf curl Yunnan virus
TGMV	Tomato golden mosaic virus
BSCTV	Beet severe curl top virus
