# Supplementary material for: A Rapid and Sensitive Loop-Mediated Isothermal Amplification Assay for the Detection of Mulberry Mosaic Dwarf-Associated Virus
Source: Biosensors (Basel). 2026 Sep 10;16(9):509. doi: 10.3390/bios16090509 (PMC13604728; doi:10.3390/bios16090509)
Supplement: Supplementary file 1 [file biosensors-16-00509-s001.zip › biosensors-4474742-supplementary.pdf]

biosensors MDPI

Supplementary Materials

A Rapid and Sensitive Loop-Mediated Isothermal Amplification Assay for the Detection of Mulberry Mosaic Dwarf-Associated Virus

Shaoshuang Sun [1,2], Xueping Zhou [2,3,*] and Xiuling Yang [2,*]

[1] Shandong Key Laboratory of Wetland Ecology and Biodiversity Conservation in the Lower Yellow River, School of Life Sciences, Qufu Normal University, Qufu 273165, China; sunshaoshuangsss@163.com (S.S.)
[2] State Key Laboratory for Biology of Plant Diseases and Insect Pests, Institute of Plant Protection, Chinese Academy of Agricultural Sciences, Beijing 100193, China
[3] State Key Laboratory of Rice Biology and Breeding, Institute of Biotechnology, Zhejiang University, Hangzhou 310058, China
* Correspondence: zzhou@zju.edu.cn (X.Z.); yangxiuling@caas.cn (X.Y.)

Table S1. Primers used in this study.

System	Primer	Sequence （5'- 3'）	Length
LAMP	FIP (F1C+F2)	GACTCGAACAGTCCTGGGCCT-CTGAGATCAAGGCGGGGA	39
	BIP (B1C+B2)	GTTGCAAGTGCCATGCGAACC-GGCCGTCTCTTCCTTCTTG	40
	F3	TGATTACCAGGAGCTCTGCT	20
	B3	GTTTCACTGGGCCCAACG	18
	LF	CCGTGGAATGGTTCCTGGATTC	22
	LB	CGTGGAGAAGAAGAGGCCCAAG	22
PCR	MCP746f	CGAGTTTGGCAAGAAGGAAGAG	22
	MCP1148r	TTGGCTCCCACTAAATGAAAGG	22

Table S2. Geminiviruses used for sequence alignment in Figure 1.

Virus	Genus	Accession Number
Mulberry mosaic dwarf-associated virus (MMDaV）	*Mulcrilevirus*	KP728254
Beet curly top virus (BCTV）	*Curtovirus*	PX253982
Beet curly top Iran virus (BCTIV）	*Becurtovirus*	JQ707938)
Maize streak virus (MSV）	*Mastrevirus*	NC_001346
Opuntia virus 1 (OpV1）	*Opunvirus*	NC_055584
Turnip curly top virus (TCTV）	*Turncurtovirus*	NC_014324
Tomato apical leaf curl virus (ToALCV）	*Topilevirus*	NC_076213
Welwitschia mirabilis associated geminivirus A (WMaGVA）	*Welwivirus*	BK061149
Tomato yellow leaf curl virus (TYLCV）	*Begomovirus*	AJ489258
Tomato pseudo-curly top virus (TPCTV）	*Topocuvirus*	NC_003825
Citrus chlorotic dwarf associated virus (CCDaV）	*Citlodavirus*	MW160707
Grapevine red blotch virus (GRBV）	*Grablovirus*	OR730839
Eragrostis curvula streak virus (ECSV）	*Eragrovirus*	NC_012664
Euphorbia caput-medusae latent virus (ECmLV）	*Capulavirus*	KT214376
Juncus maritimus geminivirus 1 (JMGV1）	*Maldovirus*	NC_037066

Table S3. MMDaV isolates used for alignment of V2 nucleotide sequences.

MMDaV Isolates	Accession Number	Percent Identity
AK2-18	KP728254	100.00%
AK2-14	KP699130	100.00%
AK1-8	NC_026771、KP303687	99.78%
AK2-38	KP699131	99.78%
AK3-54	KP699132	99.78%
AK1-3	KP699128	99.78%
AK1-4	KP699129	99.56%
ZJTX	MN240483	95.11%
